# *N^ω^*-(Carboxymethyl)arginine Is One of the Dominant Advanced Glycation End Products in Glycated Collagens and Mouse Tissues

**DOI:** 10.1155/2019/9073451

**Published:** 2019-09-10

**Authors:** Sho Kinoshita, Katsumi Mera, Hiroko Ichikawa, Satoko Shimasaki, Mime Nagai, Yuki Taga, Katsumasa Iijima, Shunji Hattori, Yukio Fujiwara, Jun-ichi Shirakawa, Ryoji Nagai

**Affiliations:** ^1^Laboratory of Food and Regulation Biology, Graduate School of Agriculture, Tokai University, Kumamoto, Japan; ^2^Department of Biopharmaceutics, Graduate School of Pharmaceutical Sciences, Kumamoto University, Kumamoto, Japan; ^3^Department of Food and Nutrition, Laboratory of Nutritional Science and Biochemistry, Japan Women's University, Tokyo, Japan; ^4^Nippi Research Institute of Biomatrix, Tokyo, Japan; ^5^Department of Cell Pathology, Graduate School of Medical Sciences, Kumamoto University, Kumamoto, Japan

## Abstract

Advanced glycation end products (AGEs) accumulate in proteins during aging in humans. In particular, the AGE structure *N^ω^*-(carboxymethyl)arginine (CMA) is produced by oxidation in glycated collagen, accounting for one of the major proteins detected in biological samples. In this study, we investigated the mechanism by which CMA is generated in collagen and detected CMA in collagen-rich tissues. When various protein samples were incubated with glucose, the CMA content, detected using a monoclonal antibody, increased in a time-dependent manner only in glycated collagen, whereas the formation of *N^ε^*-(carboxymethyl)lysine (CML), a major antigenic AGE, was detected in all glycated proteins. Dominant CMA formation in glycated collagen was also observed by electrospray ionization-liquid chromatography-tandem mass spectrometry (LC-MS/MS). During incubation of glucose with collagen, CMA formation was enhanced with increasing glucose concentration, whereas it was inhibited in the presence of dicarbonyl-trapping reagents and a metal chelator. CMA formation was also observed upon incubating collagen with glyoxal, and CMA was generated in a time-dependent manner when glyoxal was incubated with type I–IV collagens. To identify hotspots of CMA formation, tryptic digests of glycated collagen were applied to an affinity column conjugated with anti-CMA. Several CMA peptides that are important for recognition by integrins were detected by LC-MS/MS and amino acid sequence analyses. CMA formation on each sequence was confirmed by incubation of the synthesized peptides with glyoxal and ribose. LC-MS detected CMA in the mouse skin at a higher level than other AGEs. Furthermore, CMA accumulation was greater in the human aorta of older individuals. Overall, our study provides evidence that CMA is a representative AGE structure that serves as a useful index to reflect the oxidation and glycation of collagen.

## 1. Introduction

Incubation of proteins with glucose leads to the generation of advanced glycation end products (AGEs) from the Maillard reaction through the formation of Schiff bases and Amadori products [1]. The levels of AGE-modified proteins increase during the normal aging process [2]; however, this increase is markedly accelerated in a diabetic condition with sustained hyperglycemia. *N^ε^*-(Carboxymethyl)lysine (CML), one of the major antigenic AGE structures, accumulates in several tissue proteins, including the kidneys of patients with diabetic nephropathy [3] and chronic renal failure [4], atherosclerotic lesions of arterial walls [5], amyloid fibrils in hemodialysis-related amyloidosis [6], and actinic elastosis of photoaged skin [7]. Pentosidine [8], one of the fluorescent AGE structures generated under oxidative conditions, accumulates in the blood and in long-lived tissue proteins such as collagens and lens protein. Methylglyoxal is generated through the Embden-Meyerhof and polyol pathways and reacts with proteins to form *N^ε^*-(carboxyethyl)lysine (CEL) [9] and *N^δ^*-(5-hydro-5-methyl-4-imidazolon-2-yl)-ornithine (MG-H1) [10]. Accordingly, detection of CEL and MG-H1 in biological samples is used as evidence for the modification of proteins by methylglyoxal.

Recent studies have demonstrated that AGEs are generated from glucose as well as from carbonyl compounds such as glyoxal, methylglyoxal [11], glucosone [12], and glycolaldehyde [13], which are generated from the Embden-Meyerhof pathway, glucose and lipid oxidation, and inflammation, followed by rapid reaction with proteins. In addition, many reports have suggested that these carbonyl compounds play an important role in the pathogenesis of several diseases other than diabetes mellitus. For example, Arai et al. [14] reported that 20% of schizophrenia cases showed carbonyl stress with high-plasma pentosidine and low-serum pyridoxal despite no physical complications such as diabetes mellitus and renal dysfunction. Among these psychiatric patients with carbonyl stress, several of the patients with schizophrenia were found to harbor mutations in the glyoxalase 1 (*GLO*1) gene with consequent reduction of enzymatic activity, demonstrating that carbonyl stress may also participate in the schizophrenia pathogenesis. Nonenzymatic cross-links and AGE formation in bone collagen deteriorate bone toughness, postyield properties, and ductility. Moreover, Mitome et al. [15] reported that pentosidine in bone collagen was remarkably increased in dialysis patients and was inversely correlated with the bone formation rate/bone volume ratio and mineral apposition rate. This result suggests that modification of collagen by AGEs is strongly associated with disorders of bone metabolism in dialysis patients.

*N^ω^*-(Carboxymethyl)arginine (CMA) is an acid-labile AGE structure that was discovered in the enzymatic hydrolysate of glycated collagen [16]. Subsequently, CMA was also detected in human serum by liquid chromatography-tandem mass spectrometry (LC-MS/MS), and its serum level in diabetic patients was found to be higher than that in normal subjects [17]. However, an internal standard was not used in this study, and the CMA content in tissues remains unclear because of its acid instability; thus, little is known about the CMA level in organs. Collagens are one of the major proteins in the human body and play an important role in several biological functions such as formation of the extracellular matrix, which comprises the bulk of the skin and confers strength and resiliency [18], and contributes to the mechanical properties and metabolism of bone [19]. Thus, measurement of the CMA content may be a useful tool to evaluate the degeneration of collagens. To clarify the biological significance of CMA, we investigated the mechanism by which CMA is generated in glycated collagen. Furthermore, to determine the CMA content in tissues, a preparation method for tissue samples was developed and measurement was performed using LC-MS/MS along with an internal standard.

## 2. Materials and Methods

### 2.1. Materials

Bovine collagen types I and II were purchased from Koken Co. (Tokyo, Japan), and bovine collagen types III and IV were purchased from Nitta Gelatin Co. (Osaka, Japan). Human serum albumin (HSA) was donated by the Chemo-Sero-Therapeutic Research Institute (Kumamoto, Japan) and was defatted using charcoal treatment as described by Chen [20]. Ribonuclease (RNase) A, human hemoglobin, D-glucose, glyoxal, and S-Carboxymethyl-L-cysteine (CMC) were purchased from Sigma-Aldrich (St. Louis, MO, USA). 1-Ethyl-3-(3-dimethylaminopropyl) carbodiimide hydrochloride and *N*-hydroxysulfosuccinimide were purchased from Pierce (Rockford, IL, USA). Horseradish peroxidase- (HRP-) conjugated goat anti-mouse IgG antibody was purchased from Kirkegaard Perry Laboratories (Gaithersburg, MD, USA). All other chemicals were of the best grade available from commercial sources. Standard AGEs and isotope-labeled amino acids were purchased from PolyPeptide Laboratories (Strasbourg, France) and Cambridge Isotope Laboratories Inc. (Tewksbury, MA, USA), respectively. The synthesized peptides (Table 1) were purchased from AnyGen Co. Ltd. (Gwangju, Korea).

### 2.2. Preparation of CMA

CMA was synthesized by the reaction of l-arginine and glyoxal, as described by Odani et al. [17] with minor modifications. In brief, l-arginine (100 mM) was incubated with glyoxal (100 mM) in a 1.0 M sodium hydrate solution for 1 week at 37°C, since approximately 50% of the l-arginine, estimated using the peak area obtained from ESI-LC-MS/MS analysis, was unmodified after the 2-day incubation. The mixture was then neutralized with 1.0 M hydrochloric acid. The reaction products were applied to a Dowex 50W (100-200 mesh, H1 form) column (30 × 170 mm) equilibrated with distilled water, and CMA was then eluted with 10% pyridine. The eluate was evaporated to yield a residue, which was chromatographed on a silica gel column (20 × 200 mm) with 100% methanol. The fraction containing CMA was subjected to MS and nuclear magnetic resonance (NMR) measurements to confirm its structure. [^13^C_6_] CMA was also synthesized from [^13^C_6_] arginine (Cambridge Isotope Laboratories Inc., Tewksbury, MA, USA).

### 2.3. In Vitro Peptides and Protein Modification

Type I collagen (1.5 mg/mL) was incubated with 200 mM glucose in a 100 mM sodium phosphate buffer (pH 7.4) at 37°C for up to 8 weeks. The same experiment was conducted with HSA, RNase, hemoglobin, immunoglobulin, and low-density lipoprotein (LDL). Gelled collagenous samples were added to a disruption tube with 6.0 mm stainless steel beads and homogenized on a Shakemaster (Biomedical Science, Japan), followed by determination of protein concentration using a bicinchoninic acid assay (Pierce, Rockford, IL, USA). The CMA or CML content of the samples was determined by enzyme-linked immunosorbent assay (ELISA) [21] and instrumental analysis [22, 23] as described below. Ribose gelatin was also prepared by incubating 30 mM ribose with gelatin (2 mg/mL) in a 200 mM sodium phosphate buffer (pH 7.4) at 37°C for 1 week. Furthermore, each synthesized peptide (2 mg/mL) described in Table 1 was also incubated with 1 mM glyoxal or 30 mM ribose in a 200 mM sodium phosphate buffer (pH 7.4) at 37°C for 1 week.

### 2.4. Elisa

ELISA was performed as described previously [21] using the previously described monoclonal anti-CMA antibody [24]. In brief, each well of a 96-well microtiter plate was coated with 100 *μ*L of the indicated concentration of sample in PBS and incubated for 2 h. The wells were washed three times with PBS containing 0.05% Tween 20 (washing buffer), and then blocked with 0.5% gelatin in phosphate-buffered saline (PBS) for 1 h. After washing three times, the wells were incubated for 1 h with 100 *μ*L of monoclonal anti-CMA (3F5; 1 *μ*g/mL) and monoclonal anti-CML (6D12; 0.5 *μ*g/mL) antibodies. After washing thrice, the wells were incubated with HRP-conjugated anti-mouse IgG, followed by reaction with 1,2-phenylenediamine dihydrochloride. The reaction was terminated with 100 *μ*L of 1.0 M sulfuric acid, and the absorbance was read at 492 nm on a micro-ELISA plate reader. CMA contents were also measured by competitive ELISA. Each well of a 96-well microtiter plate was coated with 100 *μ*L of CMA-conjugated Keyhole limpet hemocyanin (KLH) in PBS and incubated for 2 h, and the wells were blocked with 0.5% gelatin in PBS for 1 h, followed by three washes with washing buffer. 60 *μ*L of 3F5 (2 *μ*g/mL) and 60 *μ*L of samples were preincubated for 1 h, and sample aliquots (100 *μ*L) were added to each well and incubated for 1 h. After three washes with washing buffer, the wells were incubated with HRP-conjugated anti-mouse IgG and developed as described above.

### 2.5. Formation of Arginine Adduct with High Concentration of Glyoxal

*N^α^*-Acetyl-L-arginine (10 mM) was incubated with different concentrations of glyoxal (0.1-100 mM) in PBS (pH 7.4) at 37°C for 2 days, and the elemental composition was analyzed by quadrupole time-of-flight mass spectrometry equipped with an electrospray ionization source (ESI-QTOF) using a compact mass spectrometer (Bruker Daltonics, Bremen, Germany). The reaction mixture (100 *μ*L) was mixed with 900 *μ*L of distilled water containing 0.1% trifluoroacetic acid (TFA). The samples were passed over a Strata-X-C column (Phenomenex, Torrance, CA, USA) as described previously [22, 23] with minor modification. Thus, the column was prewashed with 1 mL of methanol and equilibrated with 1 mL of 0.1% TFA. The final concentrations of the samples for the elemental composition analysis and qualitative analysis with positive-mode ESI-QTOF were 100 *μ*g/mL and 2 *μ*g/mL, respectively. For the elemental composition analysis with positive-mode ESI-QTOF, the capillary voltage was 4.5 kV and the ionization source temperature was 200°C. A collision-induced dissociation was performed using nitrogen as the collision gas at a pressure of 1.6 bar. Data were acquired with a stored mass range of 50–1000 (*m*/*z*). Detected ions were analyzed by SmartFormula manually (Bruker Daltonics, Bremen, Germany). For qualitative analysis with positive-mode ESI-QTOF, LC was conducted on a ZIC®-HILIC column (150 × 2.1 mm, 5 *μ*m) (Merck Millipore, Billerica, MA, USA). The mobile phase was collected using solvent A (distilled water containing 0.1% formic acid) and solvent B (acetonitrile containing 0.1% formic acid). The flow rate was 0.2 mL/min, and the column was kept at 40°C. The retention time for the *N^α^*-acetyl CMA dimer was approximately 12 min. The structure was detected by electrospray ionization and positive ion mass spectrometry. The target ion of 549.2627 ± 0.005 (*m*/*z*) was measured for the analysis of the *N^α^*-acetyl-L-arginine adduct with glyoxal in samples incubated with glyoxal.

### 2.6. Measurement of AGE Contents by ESI-LC-MS/MS

The contents of CMA and other AGEs in the samples were measured by electrospray ionization- (ESI-) LC-MS/MS using a TSQ Quantiva triple-stage quadrupole mass spectrometer (Thermo Fisher Scientific, Waltham, MA, USA) as described previously [22, 23] with minor modification. In brief, 3 *μ*L of glycated proteins (3 *μ*g protein) and 17 *μ*L of distilled water were mixed with 20 *μ*L of a 200 mM sodium borate buffer (pH 9.1) and reduced by the addition of 2 *μ*L of NaBH_4_ (1 M NaBH_4_ in 0.1 N NaOH) at room temperature for 4 h. Standard 10 pmol of [^13^C_6_] CMA and [^2^H_2_] CML (PolyPeptide Laboratories, Strasbourg, France) and 5 nmol of [^13^C_6_] lysine (Cambridge Isotope Laboratories Inc., Tewksbury, MA, USA) and [^13^C_6_] arginine (Cambridge Isotope Laboratories, Inc., Tewksbury, MA, USA) were added to the pellets, and samples were hydrolyzed with 1 mL of 6 N HCl at 100°C for 24 h. CMA is degraded through acid hydrolysis in the same manner as [^13^C_6_] CMA; thus, the amount of CMA can be quantitated based on the amount ratio (CMA and [^13^C_6_] CMA) and area ratio (233 (*m*/*z*) and 239 (*m*/*z*)) [25]. As the lysine content in collagen and albumin differs, the AGE content in glycated proteins *in vitro* was expressed as pmol AGEs/30 ng proteins. Furthermore, the lysine-derived AGEs were normalized by the lysine content, and arginine-derived AGEs were normalized by the arginine content; thus, the data are expressed as CML mmol/mol Lys and CMA mmol/mol Arg, respectively.

**Figure 1:**
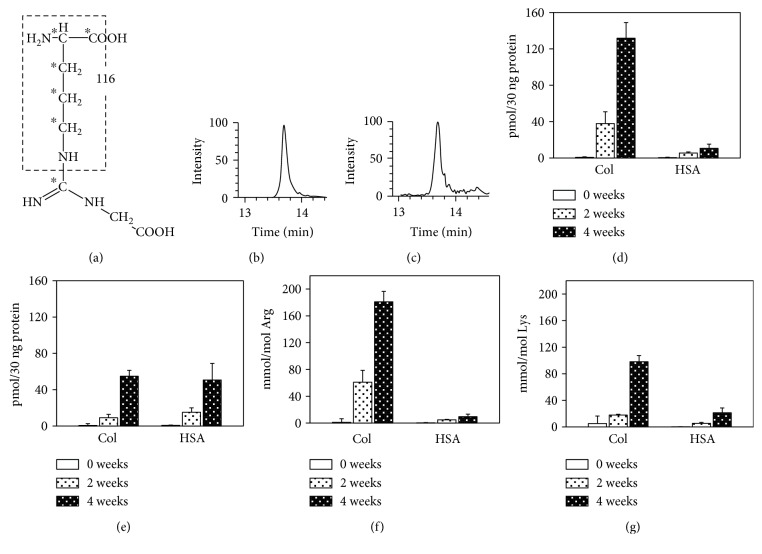
Measurement of CMA in glycated proteins by ESI-LC-MS/MS. (a) 13C in [^13^C_6_] CMA is indicated by an asterisk. Type I collagen and HSA were incubated with glucose at 37°C for up to 4 weeks. The parent ions of CMA and [^13^C_6_] CMA were 233 (*m*/*z*) and 239 (*m*/*z*), respectively, and peaks of the fragment ion of 116 (*m*/*z*) for CMA (b) and 121 (*m*/*z*) for [^13^C_6_] CMA (c) were detected in the samples of glycated collagen. The amounts of CMA (d) and CML (e) were normalized by the amounts of proteins in glycated proteins. The amount of CMA (f) was normalized by the arginine content, and CML (g) was normalized by the lysine content.

The AGE contents in mouse skin were analyzed by ESI-LC-MS/MS. All animal experiments were confirmed by Tokai University (approval number: 161076). Experiments were undertaken in compliance with the Guidelines for the Care and Use of Animals for Scientific Purposes at Tokai University (established April 1, 2007). ddy mice were purchased from Kyudo (Kumamoto, Japan) and were housed in a pathogen-free barrier facility (12 h light-dark cycle) and fed a normal rodent chow diet (CLEA Japan, Tokyo, Japan). At 20 weeks, the animals were euthanized under pentobarbital anesthesia. Skin specimens were immediately frozen and stored at −80°C until use. For measurement of AGEs, 5 mg of minced mouse skins (dry weight) and 100 *μ*L distilled water were mixed with 100 *μ*L of a 200 mM sodium borate buffer (pH 9.1) and reduced by adding 10 *μ*L of 1 M NaBH_4_ at room temperature for 4 h. The samples were centrifuged at 3,000 rpm, the supernatant was discarded, and the skin was hydrolyzed with 1 mL of 6 N HCl at 100°C for 2 h. One portion of the hydrolyzed samples (0.1 mg) was then transferred to a different tube followed by addition of the standard [^13^C_6_] CMA (Figure 1(a)), [^2^H_2_] CML, [^2^H_4_] *N^ε^*-(carboxyethyl)lysine (CEL), [^2^H_3_] *N^δ^*-(5-hydro-5-methyl-4-imidazolon-2-yl)-ornithine (MG-H1) (PolyPeptide Laboratories, Strasbourg, France), [^13^C_6_] lysine, and [^13^C_6_] arginine to the pellets, which were further hydrolyzed with 1 mL of 6 N HCl at 100°C for 22 h. The following steps were conducted in the same manner as described for the *in vitro* samples. CEL and MG-H1 of AGEs were also measured in addition to CML and CMA. The retention times for CEL and MG-H1 were 12–14 min. The parent ions of CEL and [^2^H_4_] CEL were 219 (*m*/*z*) and 223 (*m*/*z*), respectively. Fragment ions of 130 (*m*/*z*) and 134 (*m*/*z*) from each parent ion were measured for the analysis of CEL and [^2^H_4_] CEL in samples. The parent ions of MG-H1 and [^2^H_3_] MG-H1 were 229 (*m*/*z*) and 232 (*m*/*z*), respectively. Fragment ions of 114 (*m*/*z*) and 117 (*m*/*z*) from each parent ion were measured for the analysis of MG-H1 and [^2^H_3_] MG-H1 in the samples. The AGE content in mouse skin *in vivo* was expressed as nmol AGEs/*μ*g mouse skin. The lysine-derived AGEs were normalized by the lysine content, and the arginine-derived AGEs were normalized by the arginine content; the data are also expressed as CML and CEL mmol/mol Lys and CMA and MG-H1 mmol/mol Arg, respectively.

### 2.7. Isolation of CMA-Modified Peptides from Glyoxal-Modified Collagen by Affinity Chromatography

The hybridoma-producing 3F5 was inoculated into the peritoneal cavities of Balb/c mice to obtain ascetic fluid. One milliliter of protein G Sepharose (GE Healthcare, Sweden) was saturated with a 50 mM borate buffer (pH 8.2), and then 1 mL of ascites was mixed with an equal volume of borate buffer and then applied to protein G Sepharose. After the column was washed with 10 mL of borate buffer, the antibody was conjugated to Sepharose with 1 mL of 25 mM dimethyl pimelimidate (Thermo Fisher Scientific, Waltham, MA, USA) for 1 h at room temperature. The reaction was terminated by adding 0.2 M monoethanolamine (Sigma-Aldrich, St. Louis, MO, USA). To identify the CMA-modified peptides in glycated collagen, type III collagen (1.5 mg/mL) was incubated with glyoxal (1 mM) at 37°C for 1 week. Then, 0.5 mg of glyoxal collagen was further incubated at 60°C for 5 min to denature the collagen structure, followed by digestion with trypsin (10 *μ*g/mL) at 37°C for 12 h. The reaction was terminated by the addition of a 1/50 volume of a protease inhibitor cocktail (Nacalai Tesque, Kyoto, Japan), and then applied to the 3F5-conjugated affinity column. The column was washed with 10 mL of PBS, and CMA peptides were then eluted with 0.2 M glycine-HCl (pH 3.0). The adsorbed fraction was concentrated and analyzed using ESI-hybrid triple quadrupole/linear ion trap mass spectrometry (ESI-QTRAP) on a 3200 QTRAP mass spectrometer (AB Sciex, Foster City, CA, USA). The instrument was coupled to an Agilent 1200 Series HPLC system (Agilent Technologies Inc., Palo Alto, CA, USA).

### 2.8. Detection of CMA Peptide by ESI-QTRAP

The CMA-modified peptide samples isolated as described above were analyzed by ESI-QTRAP [26].

### 2.9. MS/MS Database Search

Peptide identification was accomplished using ProteinPilot software 4.0 (AB Sciex) with the Paragon™ algorithm. The acquired MS/MS spectra were searched against the UniProtKB/Swiss-Prot database (release 2011_08. 2011-07-27) for *Bos taurus* species (5857 protein entries). The search parameters included digestion by trypsin, biological modification ID focus, and 95% protein confidence threshold. We defined the confidence threshold of the identified peptides as 90%. The carboxymethylation of arginine (+58) and lysine (+58) were added to the search criteria of posttranslational modifications. The probabilities of proline and lysine hydroxylation were set higher than the defaults for collagen analysis.

### 2.10. Sequence Confirmation by a Protein Sequencer

The CMA peptide sequence was confirmed by an N-terminal amino acid sequence analysis. The tryptic digest of the glyoxal-modified type III collagen was applied to the CMA affinity column as described above. The adsorbed peptide fraction was loaded onto an Ascentis Express C18 HPLC column (Supelco, Bellefonte, PA, USA), and the CMA peptide-containing fraction was collected. This sample was analyzed by a Procise 492 protein sequencer (Applied Biosystems, Invitrogen Co., Carlsbad, CA, USA) in the pulsed liquid mode.

### 2.11. Tissue Samples and Immunohistochemical Analysis

We evaluated paraffin-embedded thoracic aortas from autopsies of 10 patients (five elderly and five young patients). Informed written consent was obtained from the families after the death of all patients, and the study design was approved by the Institutional Review Board of Kumamoto University in accordance with the World Medical Association Declaration of Helsinki. Autopsies were performed at Kumamoto University Hospital between 2000 and 2017 (approval no. 2224). After sectioning (3 *μ*M thickness), paraffin-embedded aorta tissues were used for immunostaining with anti-CMA (3F5) antibody. The sections were subsequently treated with an HRP-conjugated secondary antibody (Nichirei, Tokyo, Japan), and the reactions were visualized with diaminobenzidine.

### 2.12. Hematoxylin and Eosin (H&E) and Azan Staining

Three-micrometer-thick sections of paraffin-embedded thoracic aortas were subjected to H&E staining (Mayer's hematoxylin staining, followed by eosin Y staining) and Azan staining (Mordant, Mallory's azocarmine G solution, 5% phosphotungstic acid solution, and Mallory's aniline blue orange G stain solution) according to routine procedures.

## 3. Results

### 3.1. Immunoreactivity of Monoclonal Anti-CMA Antibody (3F5)

**Figure 2:**
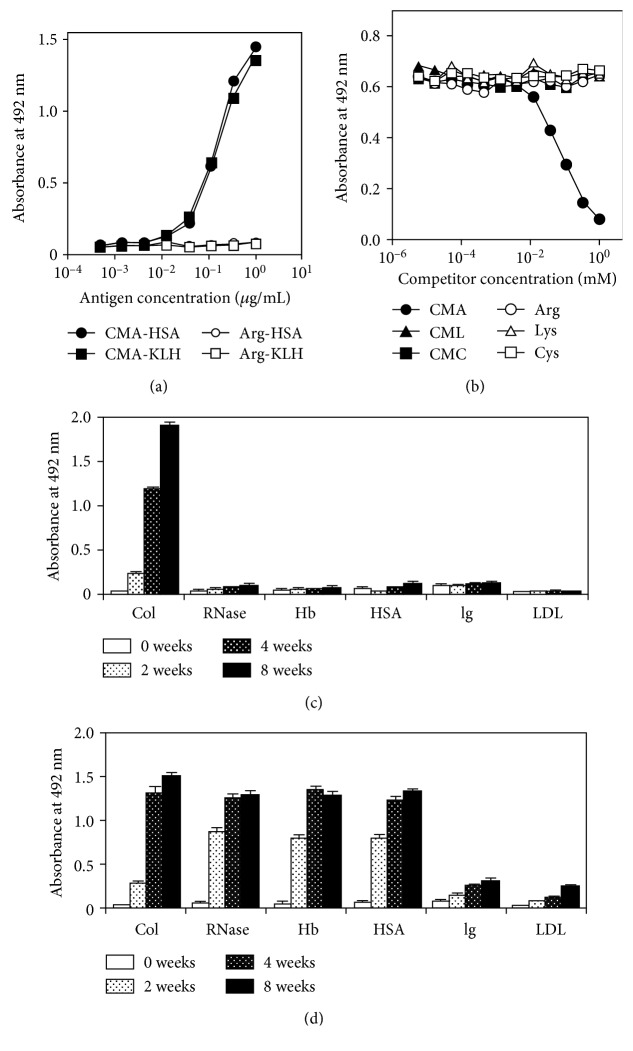
Immunoreactivity of monoclonal anti-CMA antibody (3F5). (a) Each well coated with the indicated concentration of the test sample was reacted with 3F5 (1 *μ*g/mL). The antibody bound to the wells was detected by HRP-conjugated anti-mouse IgG. (b) Specificity of the anti-CMA antibody (3F5). Each well was coated with 0.1 mL of 1 *μ*g/mL CMA-conjugated KLH and blocked with 0.5% gelatin. 60 *μ*L of 3F5 (2 *μ*g/mL) and 60 *μ*L of the samples were preincubated for 1 h, and sample aliquots (100 *μ*L) were added to each well and incubated for 1 h. The antibody bound to the wells was detected as described above. (c) CMA and (d) CML formations in glycated protein samples. Type I collagen (Col), HSA, RNase, hemoglobin (Hb), immunoglobulin (Ig), and LDL were incubated with 200 mM glucose at 37°C for up to 8 weeks, and the CMA or CML content of the samples was determined by ELISA.

The immunoreactivity of 3F5 was determined using noncompetitive and competitive ELISA. As shown in Figure 2(a), 3F5 significantly reacted with CMA-conjugated HSA and CMA-conjugated keyhole limpet hemocyanin (KLH) in a dose-dependent manner, whereas its reactivity with arginine-conjugated HSA and KLH was negligible. To examine the specificity of the antibody, we next performed competitive ELISA. Immunoreaction of 3F5 to CMA-conjugated HSA was significantly inhibited by the free form of CMA, whereas the reactivities of free CML and *S*-(carboxymethyl)cysteine (CMC) were negligible (Figure 2(b)). These results indicated that the antibody 3F5 specifically recognizes CMA.

### 3.2. CMA Formation in Glycated Proteins

CMA formation in various glycated protein samples was examined using the antibody 3F5. Glycated proteins were prepared by incubation with 200 mM glucose at 37°C for up to 8 weeks, and the CMA or CML content of the samples was then determined by ELISA. The CMA content in glycated collagen was found to increase in a time-dependent manner, whereas CMA formation was not observed in the other proteins (Figure 2(c)). In contrast, CML formation was observed in glycated collagen as well as in RNase, hemoglobin, HSA, immunoglobulin, and LDL in a time-dependent manner (Figure 2(d)).

### 3.3. CMA Detection by ESI-LC-MS/MS

As shown in Figure 1(a), the parent ions of CMA and [^13^C_6_] CMA were 233 (*m*/*z*) and 239 (*m*/*z*), respectively, and fragment ions of 116 (*m*/*z*) and 121 (*m*/*z*) were detected to measure CMA and [^13^C_6_] CMA. Fragment ion peaks of 116 (*m*/*z*) for CMA (Figure 1(b)) and 121 (*m*/*z*) for [^13^C_6_] (Figure 1(c)) were detected in the glycated collagen.

The amounts of CMA and CML were normalized by the amounts of proteins as in ELISA. As a result, the change in CMA and CML levels showed a similar tendency as detected with ELISA. Thus, although the CMA level in unmodified collagen and HSA was below detectable levels (<0.04 pmol), the CMA contents in collagens increased in a time-dependent manner, whereas the CMA levels slightly increased in glycated HSA (Figure 1(d)). The levels of CML increased by the glycation of both collagens and HSA (Figure 1(e)).

The yield of CMA was higher than that of CML (Figures 1(f) and 1(g)), and CMA was preferentially generated in collagens (Figure 1(f)). Although CML was generated both in collagens and HSA molecules, its levels in HSA were estimated to be lower than those in collagens (Figure 1(g)) given that the number of lysines differed between albumin and collagen molecules.

### 3.4. Effect of Intermediate Aldehydes on CMA Formation

**Figure 3:**
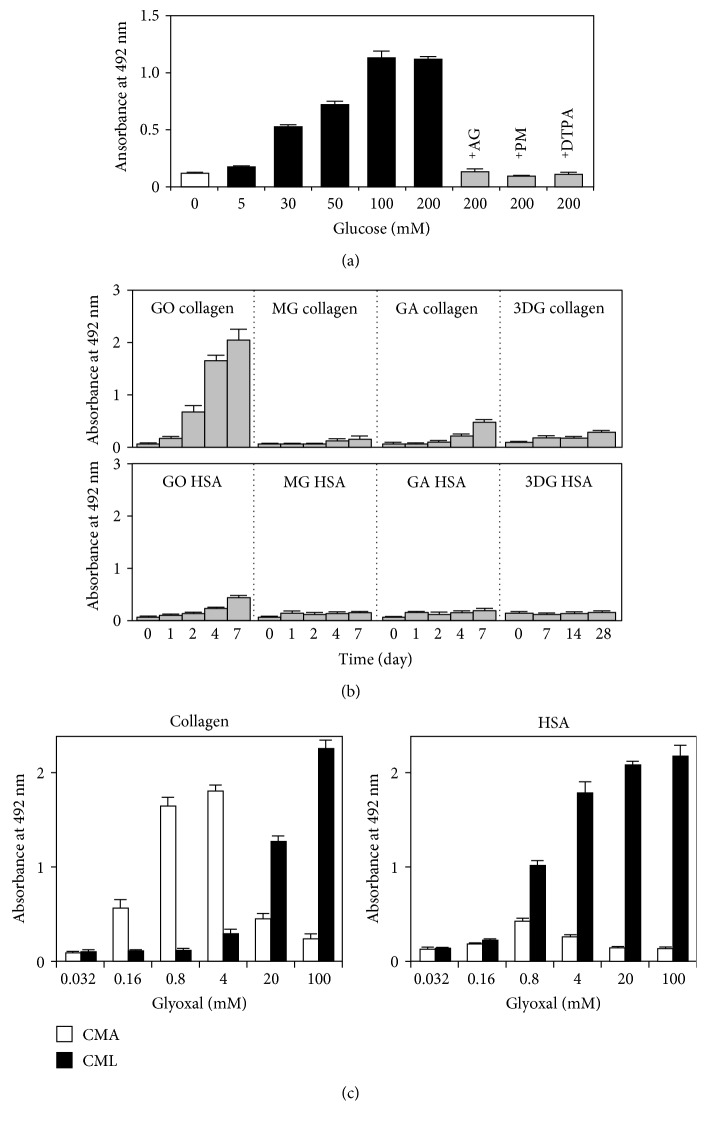
Effect of intermediate aldehydes on CMA formation. (a) Dose-dependent effect of glucose on CMA formation in glycated collagen. Type I collagen (1.5 mg/mL) was incubated with the indicated concentration of glucose at 37°C for 4 weeks. Aminoguanidine (AG), pyridoxamine (PM), and diethylenetriaminepentaacetic acid (DTPA) (1 mM each) were added before glucose addition. (b) CMA formation of aldehyde-modified collagen and HSA. Glyoxal- (GO-), methylglyoxal- (MG-), and glycolaldehyde- (GA-) modified collagen and HSA were prepared by incubation with 1 mM of these aldehydes for up to 7 days, and 3-deoxyglucosone- (3DG-) modified collagen and HSA were also prepared by incubation with 30 mM 3DG for up to 28 days. (c) Dose-dependent effect of glyoxal on CMA and CML formation in collagen. Type I collagen and HSA (1.5 mg/mL each) were incubated with the indicated concentrations of glyoxal at 37°C for 7 days, and CMA and CML contents were determined by ELISA.

The dose-dependent effect of glucose on CMA formation in glycated collagen was then measured. As shown in Figure 3(a), the CMA content increased with increasing glucose concentration and CMA formation was observed even in 30 mM glucose. This result suggested that CMA might be generated in glycated collagen under physiological glucose concentrations. Furthermore, CMA formation was significantly inhibited in the presence of the metal ion chelator diethylenetriaminepentaacetic acid (DTPA) [27] and with aldehyde-trapping reagents such as aminoguanidine [28–30] and pyridoxamine [31] (Figure 3(a)). These results suggested that intermediate aldehydes may play a role in CMA formation during the incubation of collagen with glucose. To investigate which intermediate aldehydes play a role in CMA formation, collagen was incubated with reactive aldehydes such as glyoxal, methylglyoxal, glycolaldehyde, and 3-deoxyglucosone followed by determination of the CMA content by ELISA. As shown in Figure 3(b), the yield of CMA in glyoxal-modified collagen was the highest among the aldehydes tested. In contrast, only slight CMA formation was observed in glyoxal-modified HSA (Figure 3(b)). These results indicated that glyoxal is an important precursor for CMA formation during collagen glycation.

### 3.5. Dose-Dependent Effect of Glyoxal on CMA and CML Formation in Collagen

We next examined the dose-dependent effect of glyoxal on CMA formation. Collagen and HSA were incubated with the indicated concentrations of glyoxal at 37°C for 7 days, and the CMA or CML content was determined by ELISA. As shown in Figure 3(c), the CMA content in glyoxal-modified collagen increased in a dose-dependent manner up to 4 mM glyoxal, after which it decreased in a dose-dependent manner. In contrast, the CML content increased in a dose-dependent manner up to 100 mM glyoxal in both collagen and HSA (Figure 3(c)). The incubation of *N^α^*-acetyl-L-arginine with high concentrations of glyoxal (above 10 mM) generated a parent ion of 549.2637 ± 0.005 (*m*/*z*) with an elemental composition of C_20_H_37_N_8_O_10_, possessing two CMA molecules (CMA dimer). Furthermore, we observed fragment ions of the CMA dimer: 491.2560 ± 0.005 (*m*/*z*) for the de-acetylated CMA dimer; 275.1354 ± 0.005 (*m*/*z*) for *N^α^*-acetyl-CMA; and 217.1299 ± 0.005 (*m*/*z*) for *N^α^*-acetyl-L-arginine, which demonstrated that CMA formed dimers under high concentrations of glyoxal. Interestingly, at low concentrations of glyoxal, CMA was preferentially generated in collagen over CML. These results indicate that CMA may be generated in collagen under physiological concentrations of glyoxal and that among the AGE structures determined, CMA was dominant in glycated collagen.

### 3.6. CMA and CML Formation in Different Types of Collagen (I–IV) and Heat-Denatured Type I Collagen

**Figure 4:**
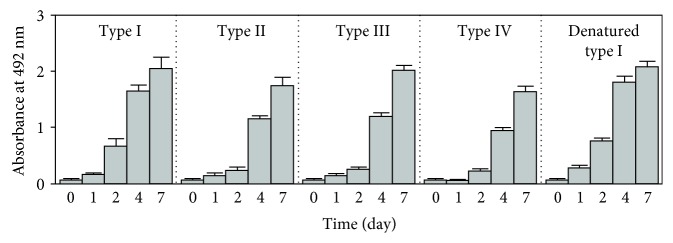
CMA formation in different types of collagen (I, II, III, and IV) and heat-denatured type I collagen. Four types of collagen (I, II, III, and IV) and heat-denatured type I collagen (denatured by heating at 60°C for 15 min) were incubated with 1 mM glyoxal at 37°C for up to 7 days, and the CMA content was determined by ELISA.

To compare CMA and CML formation in different types of collagen, several types of collagens were incubated with 1 mM glyoxal at 37°C for up to 7 days and the CMA or CML content was determined by ELISA. As shown in Figure 4, the CMA content increased time-dependently in all types of collagen, and no remarkable difference in CMA formation was observed among the collagen types. Furthermore, CMA formation in denatured type I collagen (denatured by heating at 60°C for 15 min before glyoxal incubation) was almost equal to that detected in nondenatured type I collagen (Figure 4). These results suggested that the amino acid sequence of collagen may play an important role in the CMA formation of collagen rather than the three-dimensional structure of collagen.

### 3.7. Identification of CMA Peptides in Glyoxal-Modified Type III Collagen

**Figure 5:**
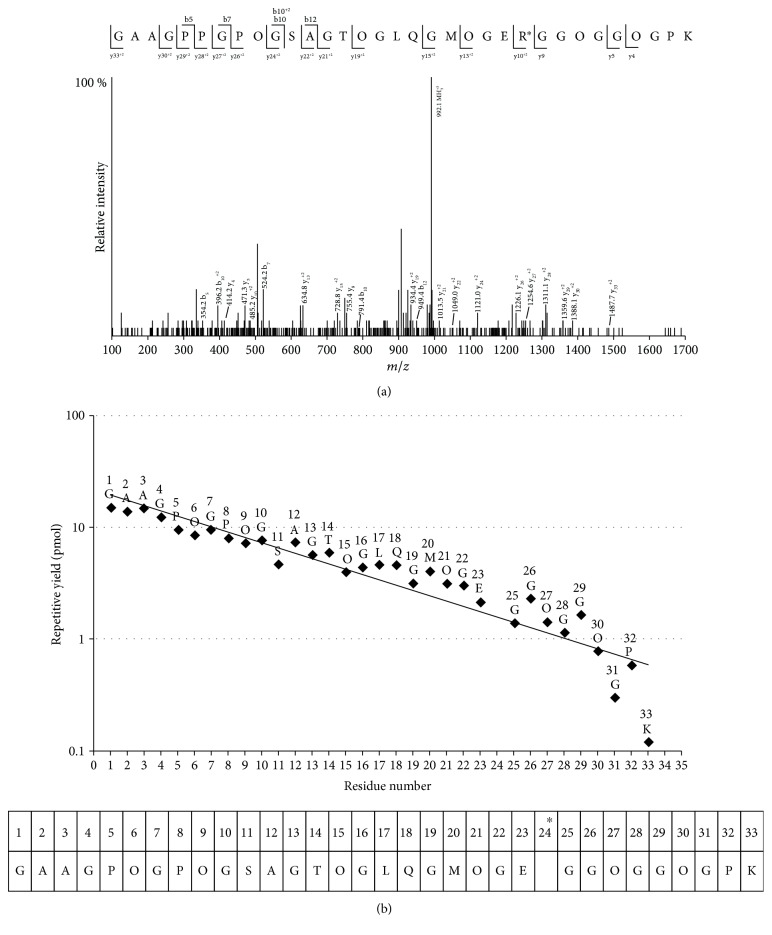
Identification of CMA peptide. (a) MS/MS spectrum of ESI-QTRAP of the CMA peptide purified with immunoaffinity column chromatography. The only CMA peptide observed after immunoaffinity purification was identified as GAAGPOGPOGSAGTOGLQGMOGER^∗^GGOGGOGPK at 992.11 (*m*/*z*) (*z* = 3^+^, O represents hydroxyproline and R^∗^ represents CMA). (b) The repetitive yield of amino acids in each step of the Edman degradation. The immunoaffinity-purified peptide was also analyzed using the 491 Protein Sequencer. The identified sequence was the same as that obtained by LC-MS/MS analysis, except for an unreadable 24th residue (asterisk).

To identify the CMA-modified peptides in glycated collagen, the tryptic digest of glyoxal-modified type III collagen was applied to the CMA affinity column, because glyoxal was found to be the dominant precursor for CMA formation (Figure 3(b)). The adsorbed peptide fraction was analyzed by ESI-hybrid triple quadrupole/linear ion trap mass spectrometry (QTRAP). As shown in Figure 5(a), one arginine-containing peptide with mass addition equivalent to carboxymethylation (+58) was detected, composed of 33 amino acids, consistent with residues 538–570 of bovine type III collagen. Next, this peptide was isolated with the same HPLC system used in ESI-QTRAP and analyzed with the 491 Protein Sequencer. Similar to the data of LC-MS/MS analysis, the sequence corresponded to residues 538–570 of bovine type III collagen, and only R561 was estimated as an unknown amino acid (Figure 5(b)), possibly because of carboxymethylation. Furthermore, the tryptic digest of glyoxal-modified type III collagen without an affinity column was also analyzed by ESI-QTRAP, and 13 peptides were detected (Table 2).

The numbering of residues begins with the triple-helical portion of the chain. The first residue corresponds to residue 15 of P04258 (type III collagen alpha 1 chain, *Bos taurus*). O represents hydroxyproline and R^∗^ represents CMA. The confidence threshold of the identified peptides was set at 90%.

### 3.8. CMA Formation in Collagen Peptides

**Figure 6:**
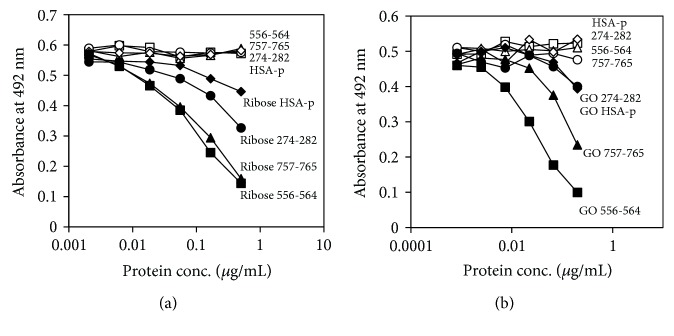
CMA formation in a collagen-like peptide. Synthesized peptides as shown in Table 1 were incubated with ribose (a) or glyoxal (b), and CMA formation was determined by competitive ELISA.

To confirm CMA formation in collagen, several collagen peptides (Table 1) were synthesized and CMA formation was measured. The sequence of bovine type III collagen corresponding to residues 538–570 was selected since this sequence was detected with or without affinity purification of glyoxal-modified collagen. The type III collagen sequences containing GXXGER motifs that are recognized by integrin were also selected, and both the N- and C-termini of four peptides composed of nine residues were coupled with (GPO)_4_ in order to form a collagen-like helical structure [32, 33]. In addition, the amount of CMA formation in these collagen peptides was compared with the HSA peptide reported as the hotspot of reactive aldehyde-modification in HSA [34]. As shown in Figures 6(a) and 6(b), CMA formation in ribose- and glyoxal-modified collagen peptides, especially in the 556-564 and 757-765 peptides, was higher than that in the HSA peptide. Furthermore, 274-282 also generated CMA, albeit weakly.

Both the N- and C-termini of the four peptides composed of nine residues were coupled with (GPO)_4_ in order to form a collagen-like helical structure.

### 3.9. Measurement of AGEs in Mouse Skin

**Figure 7:**
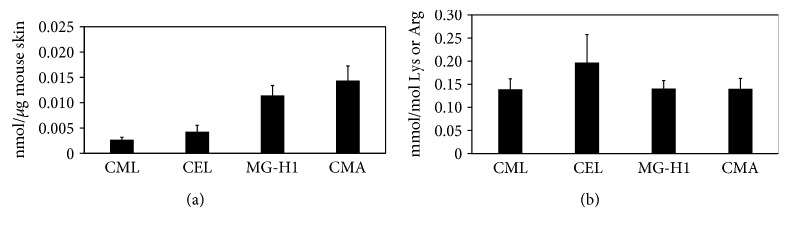
Measurement of AGEs in the mouse skin by ESI-LC-MS/MS. The AGE contents in the skin of mice (*n* = 9) were measured after hydrolysis with 6 N HCl at 100°C for 24 h. The amounts of AGEs were normalized by the dry weight (a), whereas the lysine-derived AGE contents were normalized by the lysine content and arginine-derived AGEs were normalized by the arginine content (b).

To clarify the presence of CMA in tissues, the AGE contents were measured by ESI-LC-MS/MS. As shown in Figure 7(a), the levels of CMA in the mouse skin were the highest among all measured AGEs such as CML, CEL, and MG-H1, when the AGE contents were normalized by the total amount of proteins. In particular, the level of CMA was 5-fold higher than that of CML. In contrast, when lysine-derived AGEs were normalized by the lysine content and arginine-derived AGEs were normalized by the arginine content, the level of CMA was similar to that of the other AGEs (Figure 7(b)).

### 3.10. CMA Accumulation in the Human Aorta

**Figure 8:**
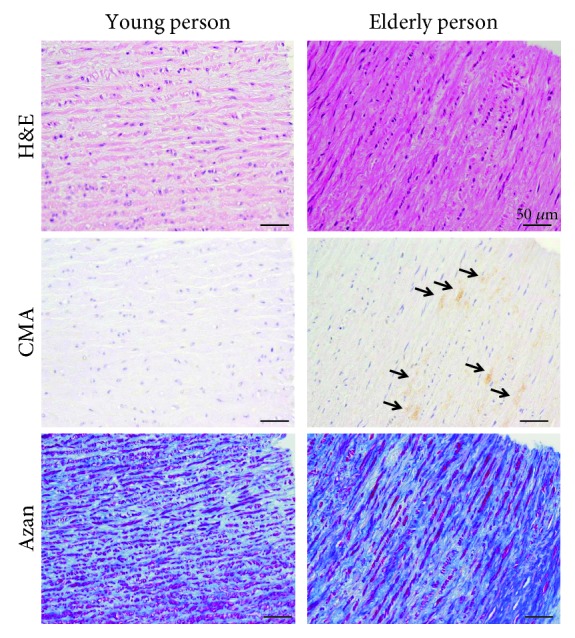
Immunohistochemistry and Azan staining of the human aorta. CMA accumulation was investigated by immunohistochemical analysis using autopsy samples from both young and elderly patients. Accumulation of CMA was detected in tissue sections of the aorta by immunostaining. The arterial collagen fibers were detected by Azan staining.

We measured the CMA accumulation in human thoracic aorta tissues, which are not generally recognized inflammation sites. Interestingly, CMA accumulation was detected by immunostaining at higher levels in the samples from elderly subjects compared to those from younger subjects (Figure 8). Furthermore, the sites of CMA accumulation were detected in the collagen layer by Azan staining (Figure 8), suggesting that the CMA accumulates at the collagen site in the aorta and that CMA accumulation is correlated with aging.

## 4. Discussion

As the measurement of chemically stable AGE structures such as CML and pentosidine is comparatively easier than that of unstable AGEs, CML and pentosidine are typically used as markers for determining the AGE content *in vivo*. The discovery of CMA was delayed because of difficulties in analysis. However, unstable AGEs may possess unique features. To clarify the biological significance of CMA, we generated a monoclonal antibody against CMA (3F5) and developed a detection system using ESI-LC-MS/MS and showed that CMA was generated in collagen during incubation with glucose and that glyoxal, an intermediate aldehyde, plays a role in CMA formation. The concentration of glucose used for the production of AGE-modified proteins varies across studies. Higashi et al. produced AGE-modified proteins by incubating bovine serum albumin with 1600 mM glucose for 40 weeks [35], while Schmidt et al. used 250 mM glucose-6-phosphate [36]; in both cases, highly modified AGE proteins were obtained. We previously reported that only highly modified AGE protein prepared with a supraphysiological concentration of glucose could be recognized by scavenger receptors such as SR-A, SR-B1, and CD36 [37], demonstrating that using the physiological concentration of glucose is important to clarify the biological significance of AGEs.

It is widely believed that the nonenzymatic modification of proteins by AGEs progresses nonspecifically and that their formation correlates with the incubation period with sugars and their concentration. Therefore, human and bovine serum albumins are widely used for glycation research, since this protein is enriched in lysine and arginine and is relatively inexpensive [12, 34, 38, 39]. We previously compared the formation of AGEs by changing dicarbonyl compounds [40, 41]; however, it remained unclear whether the type of protein affects the generation of AGEs. Interestingly, in the present study, both ELISA and ESI-LC-MS/MS showed dominant CMA formation in glycated collagen, demonstrating that CMA is generated in collagens during incubation with carbohydrates.

As CML was observed in collagen and other proteins during glucose incubation, preferential CMA formation in glycated collagen could not be explained simply by the difference in the reactivity of glucose with proteins. CMA formation in denatured collagen during incubation with 1 mM glyoxal was almost equal to that in nondenatured collagen, suggesting that a collagen-like amino acid sequence may play an important role in the CMA formation of collagen. In fact, the collagen-specific sequence was identified as the hotspot of CMA formation in glycated collagen using ESI-QTRAP. Furthermore, the peptide sequence 538–570 of bovine type III collagen was synthesized because this peptide could be detected with or without affinity chromatography. Collagens are composed of unique repeated GXX sequences. In the present study, 100% of the 15 detected CMA peptides were composed of GXR^∗^ sequences (where R^∗^ represents CMA), whereas human albumin, which generates a lower amount of CMA, has only one GXR sequence. Among the GXR^∗^ sequences, the second amino acid was acidic such as glutamic acid (in peptide 277-291) and aspartic acid (in peptide 334-348) or neutral such as alanine (in peptide 226-258), phenylalanine (in peptide 318-333), or proline (in peptide 514-537), whereas the basic amino acid sequences such as GRR, GKR, and GHR were not present in the identified CMA peptides. These results indicate a lack of regularity in the carboxymethylation of the third amino acid residue (R) and the type of the second amino acid on the GXR sequence. Furthermore, the amount of CMA formation in these collagen peptides containing the GXR sequence was compared with that of the HSA peptide, which was reported as the hotspot of reactive aldehyde-modification in HSA [34]. As a result, CMA formation in the synthesized collagen peptides was significantly higher than that in HSA peptides. These hotspot regions include the GXXGER sequence, which was reported as the integrin (*α*2*β*1) recognition site [42]. These results also demonstrated that a collagen-like amino acid sequence plays an important role in CMA formation in collagen and that the difference in amino acid sequence may be a reason for the preferential generation of CMA in glycated collagen.

Dobler and colleagues [43] demonstrated that arginine residues in the RGD and GFOGER motifs on collagen sequences are modified by methylglyoxal to form a hydroimidazolone derivative. Taken together, their report and our present observation demonstrate that collagen-specific sequences are susceptible to modification by dicarbonyls.

The inhibition of CMA formation by dicarbonyl-trapping reagents during incubation of collagen with glucose and the predominant CMA formation from glyoxal demonstrate that glyoxal plays a role in generating important intermediates for CMA formation. Furthermore, CMA formation was increased with increasing glyoxal concentration but was inhibited at a concentration greater than 4 mM. In contrast, CML formation increased in accordance with the glyoxal concentration. Although CMA was detected by ELISA on collagen modified with glyoxal (1 mM), the reactivity disappeared after the sample was further incubated with 100 mM glyoxal, since supraphysiological concentrations of glyoxal formed a dimer of CMA (data not shown).

CMA formation was higher than that of CML under low concentrations of glyoxal, which was supported by our previous observation. Thus, glucose and glycolaldehyde predominantly modify lysine residues more than arginine residues, whereas glyoxal and methylglyoxal modify arginine residues more than lysine residues [40], demonstrating that CMA may be the predominant AGE formed from glyoxal *in vivo.* Therefore, to demonstrate the physiological content, CMA levels in the skin of mice were determined by ESI-LC-MS/MS. The CMA content in the mouse tissue was similar to that of other AGEs when the levels of lysine-derived and arginine-derived AGEs were normalized to lysine and arginine contents, respectively. However, the same tendency of AGE levels in glycated collagen *in vitro* was also observed in physiological samples. Thus, the level of CMA per tissue protein was the highest among the measured AGEs and was 5-fold higher than that of CML. Moreover, higher CMA accumulation was detected in the aortas from elderly subjects compared to that from aortas of younger subjects, suggesting that CMA accumulation is increased with aging in collagen-rich tissues. Taken together, our study demonstrates that CMA is generated in glycated collagen *in vitro* and in collagen-rich tissues such as the mouse skin and human aorta. Although Thornalley et al. [11] measured the AGE content in biological samples by enzymatic hydrolysis, multiple preparation steps are required in this method. In the present study, CMA levels in the mouse skin were measured after acid hydrolysis. Cotham et al. [44] reported that glyoxal generates not only CMA but also cyclic structures such as hydro-imidazolinones (G-H1, G-H2, and G-H3). Our preliminary study demonstrated that approximately 90% of the CMA was cyclized, with 6 N HCl at 100°C for 24 h. CMA content was quantitated using an internal standard based on the principle reported by Shaw et al. [25] (data not shown).

Surprisingly, since collagen produced more CML as well as CMA compared to albumin, collagens are likely to be glycated proteins. As collagens are one of the most abundant proteins and are involved in many physiologically important functions, specific detection of CMA may play an important role in the elucidation of the relationship between the glycation and denaturation of collagens.

## Figures and Tables

**Table 1:** Synthesized collagen peptides.

Protein	Position	Sequence
Collagen	556-564	(GPO)_4_**GMOGERGGO** (GPO)_4_
Collagen	757-765	(GPO)_4_**GPOGERGAO** (GPO)_4_
Collagen	274-282	(GPO)_4_**GPRGERGEA** (GPO)_4_
HSA	405-413	(GPO)_4_**NALLVRYTK** (GPO)_4_

**Table 2:** Identification of CMA peptides in glyoxal-modified type III collagen.

Position	CMA site	Sequence of identified peptide	*m*/*z*	*z*	M.W	Identified
226-258	243	K.GEMGPAGIOGAOGLIGA**R**^∗^GPOGPOGTNGVPGOR.G	778.63	4	3110.52	Day 7
277-291	279	R.GE**R**^∗^GEAGSOGIAGPK.G	486.23	3	1455.70	Day 1
297-321	315	K.DGSOGEOGANGLOGAAGE**R**^∗^GVOGFR.G	815.36	3	2443.08	Day 7
318-333	321	V.OGF**R**^∗^GPAGANGLOGEK.G	538.93	3	1613.78	Day 7
334-348	339	K.GPOGD**R**^∗^GGOGPAGPR.G	478.89	3	1433.66	Day 7
423-440	426	K.NGE**R**^∗^GGOGGOGPQGPAGK.N	560.59	3	1678.77	Day 7
514-537	525	R.GPOGAGGPOGP**R**^∗^GGAGPOGPEGGK.G	695.32	3	2082.97	Day 7
538-570	561	K.GAAGPOGPOGSAGTOGLQGMOGE**R**^∗^GGOGGOGPK.G	992.11	3	2973.34	Day 7
588-609	591	K.DGP**R**^∗^GPTGPIGPOGPAGQOGDK.G	705.67	3	2114.00	Day 7
610-630	624	K.GESGAOGVOGIAGP**R**^∗^GGOGER.G	660.98	3	1979.93	Day 7
754-786	762	K.GDSGPOGE**R**^∗^GAOGPQGPPGAOGPLGIAGLTGAR.G	1008.82	3	3023.47	Day 7
763-798	786	R.GAOGPQGPOGAPGPLGIAGLTGA**R**^∗^GLAGPOGMOGAR.G	813.16	4	3248.98	Day 1, day 7
787-807	798	R.GLAGPOGMOGA**R**^∗^GSOGPQGIK.G	669.99	3	2006.98	Day 7
813-842	822	K.OGPSGQNGE**R**^∗^GPOGPQGLOGLAGTAGEOGR.D	963.12	3	2886.35	Day 7
862-890	864	K.GD**R**^∗^GENGSPGAOGAOGHOGPOGPVGPAGK.S	667.30	4	2665.21	Day 1, day 7

## Data Availability

The data used to support the findings of this study are available from the corresponding author upon request.

## Abbreviations

AGEs: Advanced glycation end products
CMA: *N^ω^*-(Carboxymethyl)arginine
CML: *N^ε^*-(Carboxymethyl)lysine
CMC: *S*-(Carboxymethyl)cysteine
MG-H1: *N^δ^*-(5-Hydro-5-methyl-4-imidazolon-2-yl)-ornithine
CEL: *N^ε^*-(Carboxyethyl)lysine
ESI-LC-MS/MS: Liquid chromatography-tandem mass spectrometry equipped with an electrospray ionization source
ESI-QTRAP: Hybrid triple quadrupole/linear ion trap mass spectrometer equipped with an electrospray ionization source
HSA: Human serum albumin
DTPA: Diethylenetriaminepentaacetic acid.

## Supplementary Materials

Supplementary Materials Supplementary Information: formation of arginine adduct with high concentration of glyoxal.
