# Supplementary material for: Nω-(Carboxymethyl)arginine Is One of the Dominant Advanced Glycation End Products in Glycated Collagens and Mouse Tissues
Source: Oxid Med Cell Longev. 2019 Sep 10;2019:9073451. doi: 10.1155/2019/9073451 (PMC6754957; doi:10.1155/2019/9073451)
Supplement: Supplementary Materials — Supplementary Information: formation of arginine adduct with high concentration of glyoxal. [file 9073451.f1.pdf]

Supplementary Information

a

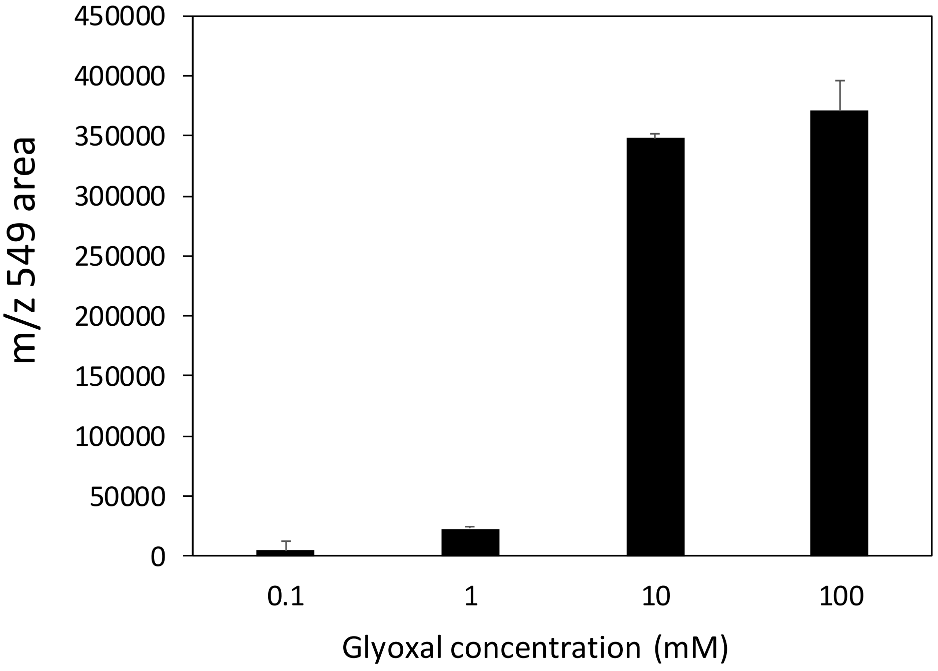

b

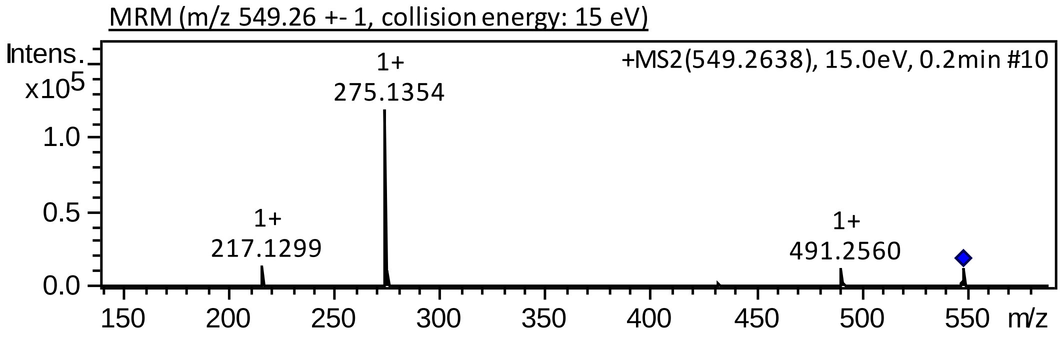

Formation of arginine adduct with high concentration of glyoxal. (a) The incubation of N^{α}-acetyl-L-arginine with high concentrations of glyoxal (above 10 mM) generated parent ion of 549.2637± 0.005 (*m/z*) with elemental composition of $C_{20}H_{37}N_8O_{10}$ that possesses two CMA molecules (CMA dimer). N=3. (b) Fragment ions of CMA dimer: 491.2560± 0.005 (*m/z*) for de-acetylated CMA dimer, 275.1354± 0.005 (*m/z*) for N^{α}-acetyl-CMA, and 217.1299± 0.005 (*m/z*) for N^{α}-acetyl-L-arginine.
